# Bronchus Associated Lymphoid Tissue Lymphoma Presenting with Immunodeficiency and Multiple Pulmonary Nodules

**DOI:** 10.1155/2017/4804378

**Published:** 2017-03-13

**Authors:** Sermin Borekci, Murat Ozbalak, Ezel Ersen, Hilal Akı, Muhlis Cem Ar, Sema Umut

**Affiliations:** ^1^Pulmonology Department, Istanbul University Cerrahpasa Medical Faculty, Istanbul, Turkey; ^2^Internal Medicine Department, Istanbul University Cerrahpasa Medical Faculty, Istanbul, Turkey; ^3^Thoracic Surgery Department, Istanbul University Cerrahpasa Medical Faculty, Istanbul, Turkey; ^4^Pathology Department, Istanbul University Cerrahpasa Medical Faculty, Istanbul, Turkey; ^5^Hematology Department, Istanbul University Cerrahpasa Medical Faculty, Istanbul, Turkey

## Abstract

Bronchus Associated Lymphoid Tissue Lymphoma (BALTOMA) is a rare subgroup of pulmonary non-Hodgkin's lymphomas (NHLs) comprising less than 1% of all cases. It constitutes 3.6% of all extranodal lymphomas and only 0.5–1% of primary pulmonary malignancies. They are usually low grade B-cell lymphomas and are considered to originate from the mucosa associated lymphoid tissue (MALT) of the bronchi. Here, we represent a rare case of BALTOMA presenting with immunodeficiency and multiple pulmonary nodules.

## 1. Introduction

Bronchus Associated Lymphoid Tissue Lymphoma (BALTOMA) is a rare subgroup of pulmonary non-Hodgkin's lymphomas (NHLs) comprising less than 1% of all cases. It constitutes 3.6% of all extranodal lymphomas and only 0.5–1% of primary pulmonary malignancies [1]. They are usually low grade B-cell lymphomas and are considered to originate from mucosa associated lymphoid tissue (MALT) of bronchi [2]. Lung is one of the most frequent nongastrointestinal sites where MALT lymphoma, also called BALTOMA, originates [3, 4]. These lesions were described as “pseudolymphoma” in the past due to their relatively indolent course and bland histological appearances [5]. Here we represent a rare case of BALTOMA presented with immunodeficiency and multiple pulmonary nodules.

## 2. Case Report

**Figure 1:**
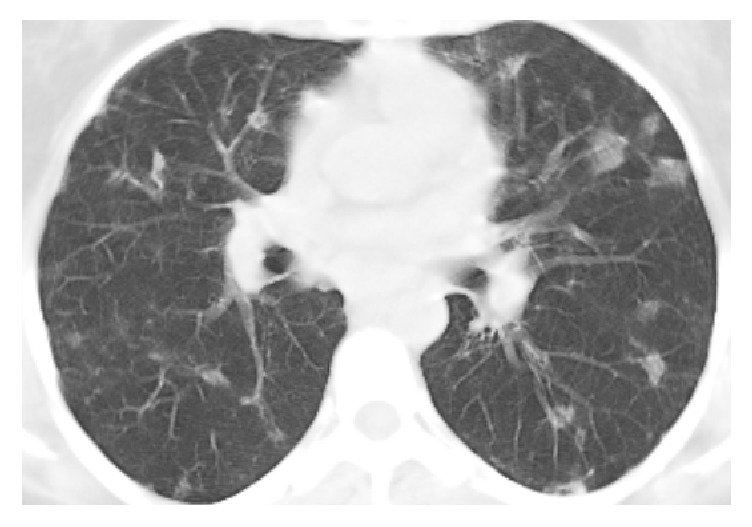
Thoracic computerized tomography showed bilateral, irregularly bordered, multiple ground-glass pulmonary nodules with diameters ranging from a few millimeters to 20 mm and, additionally, patchy ground-glass areas were detected.

A 42-year-old woman presented to our clinic in March 2015 with complaints of fatigue and having frequent flu-like episodes. Fatigue was present for 3 years and it progressed during last 3 months. She had had recurrent upper and/or lower airway infections almost every month during the previous year and had used several antibiotic agents. She had 20-pack-year history of smoking cigarettes and she had no additional comorbidities. Physical examination revealed only splenomegaly without any particular finding. Complete blood count and liver and renal function tests were totally within normal limits. Erythrocyte sedimentation rate was normal, and C-reactive protein was increased (16.2 mg/L; normal range: <5 mg/L). The chest X-ray and thoracic computerized tomography (CT) (Figure 1) showed bilateral, irregularly bordered, multiple ground-glass pulmonary nodules with diameters ranging from a few millimeters to 20 mm and additionally, patchy ground-glass areas were detected.

**Figure 2:**
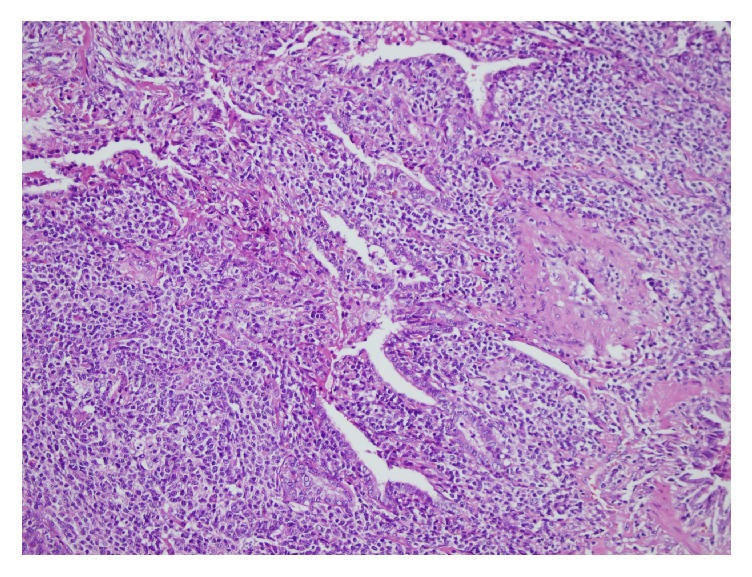
The lymphocytes infiltrated bronchiolar epithelium forming well-defined lymphoepithelial lesions. Scattered germinal centers were present throughout (hematoxylin-eosin staining, ×200).

**Figure 3:**
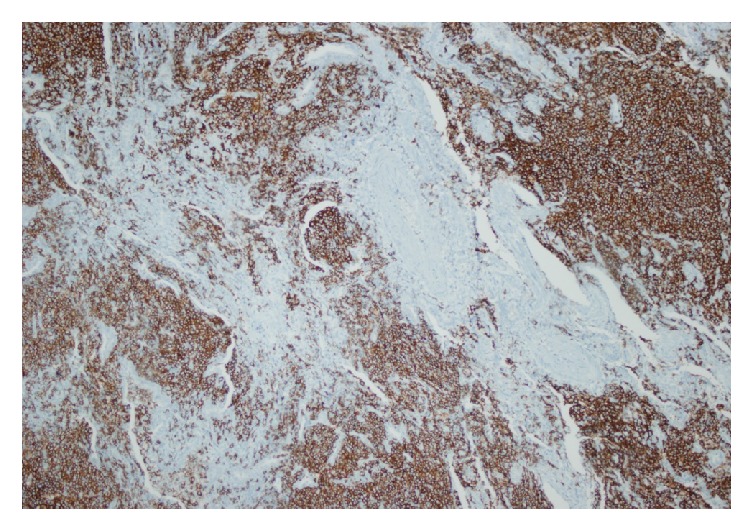
CD20 positive B lymphocytes.

**Figure 4:**
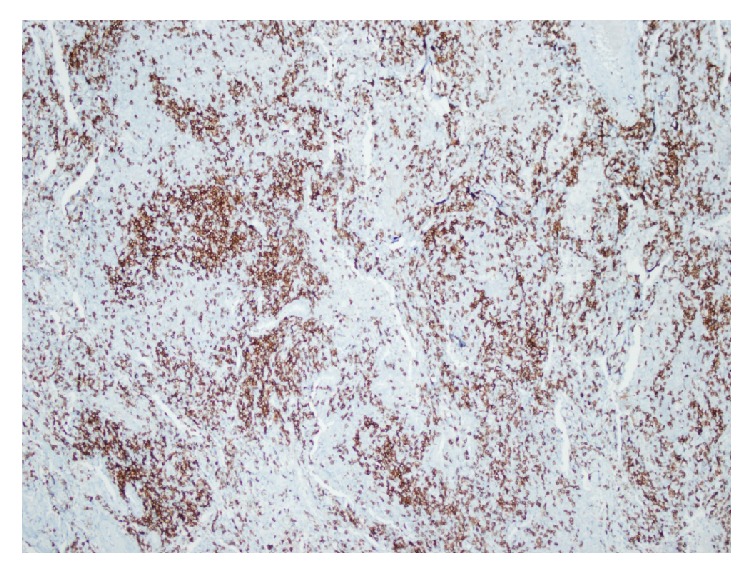
The cells were negative for CD5.

Pulmonary function test values were as follows: FEV_1_/FVC: %92, FVC: 3120 mL (84%), FEV_1_: 2860 mL (89%), and DLCO: 56%. Infections, immune deficiency syndromes, sarcoidosis, vasculitis, pulmonary involvement of collagen tissue diseases, malignancies, and metastatic disease were possible diagnostic alternatives. Our patient was hypogammaglobulinemic; serum total protein level was low (4.8 gr/dL; normal range: 6.4–8.3 gr/dL) with normal albumin level and also serum total Ig G (0.1 mg/dL; normal range: 70–400 mg/dL) and IgA (40 mg/dL; normal range: 700–1600) levels were decreased. Tuberculin skin test result was “0 mm” and ANA, anti-dsDNA, cANCA, pANCA, RF, and anti-CCP antibodies were not detected. Serologic test for Human Immunodeficiency Virus was negative. Serum procalcitonin level was within normal limits. Fundoscopic examination, echocardiography, and bronchoscopic evaluation did not reveal any additional findings. No acid fast bacilli were detected in bronchoalveolar lavage (BAL) fluid, all microbiological cultures were negative, and CD4/CD8 ratio was 0.22 in BAL fluid (lymphocyte: 25%, neutrophil: 20%, macrophage: 53%, and eosinophil: 2%). Serum and BAL angiotensin converting enzyme (ACE) and adenosine deaminase (ADA) values were within normal limits. Our patient underwent limited thoracotomy and wedge biopsy (from lingula, superior segment, and basal segment of left lower lobe). Histopathologic examination revealed multifocal nodules composed of small centrocyte-like lymphocytes with a rather uniform appearance, slightly indented nuclei, and a moderately sized pale cytoplasm. Nucleoli were inconspicuous and mitotic figures were rare. The lymphocytes infiltrated bronchiolar epithelium forming well-defined lymphoepithelial lesions. Scattered germinal centers were present throughout (Figure 2). B lymphocytes expressing CD20 and moderate intensity of IgM kappa surface immunoglobulin were shown in Figure 3. The cells were negative for CD5, CD10, CD21, and all T-cell-associated markers studied (Figure 4). Multifocal low grade B-cell lymphoma of MALT type was finally diagnosed.

Bone marrow biopsy was immediately performed to determine the stage of the disease.

Bone marrow was not infiltrated. Since PET/CT scan revealed increased FDG uptake (SUVmax value minimum: 6.48, maximum: 21.45) within all pulmonary nodules, mediastinal lymph nodes, bilateral axillar, inguinal, internal, and external iliac lymph nodes and also within the spleen the disease was defined as stage IV. Rituximab-based chemotherapy was planned; however, our patient, having some concerns about chemotherapy, did not decide about her treatment and meanwhile, because of moving to another city due to professional reasons, she was lost to follow-up.

## 3. Discussion

MALT lymphomas have been first described in 1983 by Isaacson and Wright. They have been recognized as a separate entity, accounting for approximately 8% of all NHLs [6]. Lung is the most frequent nongastrointestinal organ involved by MALT lymphoma [4].

BALTOMAs are rare and often recognized on routine chest radiographs of middle-aged patients since they produce minimal symptoms [4, 7]. Patients may have cough, chest pain, hemoptysis, and dyspnea. Systemic symptoms of lymphoma such as fever, night sweats, and weight loss may be present [4, 8]. In our case, only two complaints were present: fatigue and frequent infections. Fatigue is an expected complaint and frequent infections can also be seen in BALTOMA patients due to immune compromise [4], hypogammaglobulinemia in our case.

Chest X-rays of BALTOMA patients are usually nondiagnostic: solitary nodules (23%), multiple nodules (32%), air-space consolidation with air bronchogram (18%), patchy air-space and/or interstitial infiltrate (23%), peribronchial thickening (9%), hilar or mediastinal lymphadenopathy (5%), and pleural effusion (9%) were reported [4, 7]. We should keep in mind that there are many conditions that could have these radiologic features and none of them are diagnostic. Multiple nodules and patchy ground-glass areas were present in radiological images of our patient.

To diagnose BALTOMA, simple needle biopsy is often inadequate; thoracoscopic or limited thoracoscopic biopsy is required to have an adequate tissue sampling. The diagnosis occasionally has been achieved by less aggressive biopsies (bronchial, transbronchial, and transthoracic) [4]. We also preferred limited thoracotomy and wedge biopsy (from lingula, superior segment, and basal segment of left lower lobe) to acquire adequate tissue material. However, in contrast to the conventional approach, Borie and his colleagues reported that most of their patients (71.4%) with multilobar or disseminated disease were diagnosed by minimally invasive procedures, such as fibreoptic bronchoscopy, bronchial and transbronchial biopsies, and CT-guided percutaneous transthoracic biopsies [8], supporting that minimally invasive biopsy procedures might be preferred in selected cases.

The genotyping studies may be useful in diagnosis of BALTOMA. Nicholson et al. studied tissue species of 45 lymphoma patients (39 primary pulmonary B-cell lymphomas, 3 multifocal lymphomas that arose in the lung and at another extranodal site, and 3 that had spread to the lung from another extranodal mucosal site) that were selected among 1958 non-Hodgkin's lymphoma cases and showed that when morphological features and modern techniques such as immunohistochemistry and genotyping studies in tissue specimens are used together, the diagnosis of pulmonary lymphomas can be made more confidently [9].

Many treatment options like surgical excision, surgery followed by radiotherapy, or chemotherapy exist but optimal therapy remains unclear because of the absence of prospective studies. Surgical resection in combination with chemotherapy or radiotherapy was the main treatment in most series with favorable response rates. Rituximab treatment can also be used alone or combined with chemotherapy [4]. One of the largest series published by Stefanovic et al. showed favorable prognosis of BALTOMA with an overall survival of 95% at 80 months [10]. Cordier et al. reported an overall survival of 100% at 2 years and 94% at 5 years in 51 BALTOMA patients. Sixty-nine percent of them were treated with surgery alone or in combination with chemotherapy or radiotherapy [3]. However, surgery and localized radiotherapy might be options only for localized disease and advanced stage BALTOMA should be treated by combination chemotherapy. Rituximab-based combination chemotherapy was planned to treat our BALTOMA case, regarding patient's age, performance, comorbidities, and preferences.

In conclusion, the clinical and imaging features of primary pulmonary lymphomas are clues for the diagnosis. BALTOMA should be kept in mind in the differential diagnosis of patients with multiple pulmonary nodules and immune compromise.
